# Quantifying the effects of training in lung transplantation: Lessons from NASA

**DOI:** 10.1016/j.jhlto.2024.100102

**Published:** 2024-04-30

**Authors:** Nicholas J.S. Chilvers, Zachariah M. Evans, Alexander W. Clark, Muhammad I. Mydin, Stephen C. Clark

**Affiliations:** Cardiothoracic Centre, Freeman Hospital, Newcastle upon Tyne NE7 7DN, UK

**Keywords:** transplantation, lung, education, patient safety, thoracic surgery

## Abstract

**Background:**

Sequential lung transplantation is a complex procedure. The traditional training model continues to center around operative experience with progressively increasing delegation of stages of the procedure. However, little evidence exists on the effects this has on surgical trainers. The NASA-TLX is a subjective, multidimensional assessment tool that rates perceived workload. We sought to employ this as a means of assessing the impact that training in lung transplantation has on trainers.

**Methods:**

We prospectively collected the NASA-TLX data for 60 patients undergoing bilateral sequential lung transplantation. In 30 cases, the operation was performed entirely by the senior surgeon (SS) who implanted both lungs. In 30 operations, the senior surgeon implanted the right lung (SSR) and supervised a trainee implanting the left lung (TL).

**Results:**

The overall weighted rating was significantly lower for the surgeons undertaking the case themselves rather than training (*p* < 0.001). Cases were comparable in terms of case type, donor ischemic time, and peri-operative characteristics. Mental and temporal demands were greater throughout training cases (*p* < 0.001, *p* < 0.05). There was less effect on frustration, physical demand, and effort. Perceived performance showed no significant difference between the groups.

**Conclusions:**

The NASA task load index can be used to inform the effects of training in lung transplantation on trainers. Training leads to greater mental and temporal demands with less effect on other factors. Crucially, there was no significant difference in perceived performance. As the specialty continues to be confronted with diverse challenges, this study should give confidence to those training the transplant surgeons of the future, as well as provide them with a mechanism to reflect on their own training performance.

**ACGME Competencies:**

Interpersonal and communication skills.

Practice-based learning and improvement.

## Background

Sequential lung transplantation is a complex procedure and is challenging to teach to technically inexperienced surgeons in training. Despite many advances, it remains a practically demanding, high-risk procedure^1^ that is frequently performed out of hours^2^ and relies on a limited supply of donor organs. This in turn limits high volume and consistent exposure to cases for training. Hence, experience is most often gained through fellowships after completion of cardiothoracic training typically, in the UK, 18 months in duration.^2^

The traditional training model continues to center around operative experience with progressively increasing delegation of stages of the procedure. However, there is little evidence of the effects this has on surgical trainers. The National Aeronautics and Space Administration Task Load Index (NASA-TLX – NASA, Mountain View, CA) is a subjective, multidimensional assessment tool that rates perceived workload to assess a task, system, or team's effectiveness or other aspects of performance.^3^ We sought to employ this as a means of assessing the impact that training has on trainers, drawing comparison to procedures in which a senior surgeon (SS) is the first operator throughout. To our knowledge, this is the first description in the literature of the NASA-TLX being utilized for this purpose.

## The NASA-TLX and its application in medicine

The NASA-TLX was developed in NASA's Ames Research Center by the Human Performance Group following extensive research involving over 40 laboratory simulations.^4^ It is the most commonly used method for assessing mental workload,^5^ cited in over 4400 studies^6^ and employed in a variety of domains including aviation, driving, and health care.^7^ The method also continues to be modified by research groups to increase its relevance to particular tasks or situations.^7^

Total workload is divided into 6 subjective subscales of mental, physical, and temporal demands together with performance, effort, and frustration.^3^ The user must assign a score to each of the 6 domains in turn. The second part of the TLX creates an individual weighting of each of these subscales by asking the subject to compare them pairwise. For each comparison, the subject selects the subscale perceived to be more important for that particular task or activity. This process generates a score for each of the 6 factors and the overall weighted score for that task,^3^ allowing a more complex analysis than other simpler measures. Although originally designed to be completed with pencil and paper, the NASA-TLX is now available as a user-friendly mobile “App.”^8^

The NASA-TLX has been employed across many aspects of health care and a recent review found it to be the most commonly applied tool to assess intraoperative cognitive workload.^9^ Indeed, 1 group has even devised a specific “SURG-TLX.”^10^ The NASA-TLX has been used to assess surgical training^11^, ^12^, ^13^, ^14^ and to compare different surgical approaches,^15^, ^16^ specialties,^17^ equipment,^18^, ^19^ and intra-operative roles.^20^, ^21^ Furthermore, it has demonstrated that cognitive workload is affected by the length of surgery,^20^, ^22^ distractions,^23^, ^24^ blood loss,^22^ and unexpected procedural difficulty.^17^ In cardiothoracic surgery specifically, it has demonstrated that cognitive workload is affected by cardiopulmonary bypass,^25^, ^26^ the extent of which varies by job role and length of surgery.^26^ It has also been used to compare different thoracic approaches,^27^, ^28^ for example, robotic vs thoracoscopic,^28^ and novel surgical planning techniques.^29^

## Patients and methods

We prospectively collected the NASA-TLX data for patients undergoing bilateral sequential lung transplantation to assess the effects of training on the trainer over the course of 3 years. The procedure was either performed by the SS throughout, who implanted both lungs, or as a training case with the SS implanting only the right lung and a surgical trainee implanting the left side under supervision in the same recipient. All cases were consecutive for the supervising surgeon, randomized, and performed on cardiopulmonary bypass according to the unit's routine practice in a standard repeatable fashion. All patients on the unit's waiting list were included as potential recipients in the study and all patients are routinely consented for the purposes of being operated upon by surgeons in training under appropriate supervision. No ethical permission was required for this study as the study subject was the training surgeon and not patients. The SS remained the same for all cases and all trainees were post-certificate of completion of training fellows of similar experience. Sixty patients underwent bilateral lung transplantation. With the exception of median recipient age, there were no differences in recipient characteristics, donor ischemic time, or peri-operative parameters between groups(Table 1). There were no differences in whether surgery took place between daytime and night accepting that the sample size of cases is relatively small.

**Table 1:** Patient and Peri-Operative Characteristics for SS and SSR Patient Groups

Variable	SS group	SSR group	*p*-value
Number of transplants	30	30	
Donor age median (IQR)	51 (41-64)	46 (34-55)	0.06
Donor cause of death			
CVA/ICH	22 (73%)	24 (80%)	0.82
RTA	3 (10%)	2 (7%)	0.95
Other trauma	1 (3%)	2 (7%)	0.95
Other	4 (13%)	2 (7%)	0.75
Donor CMV status			
Negative	18 (60%)	17 (57%)	0.85
Positive	12 (40%)	13 (43%)	0.85
Donor smoking history			
No	18 (60%)	17 (57%)	0.85
Yes	12 (40%)	13 (43%)	0.85
Recipient age at transplant median (IQR)	55 (40-61)	62 (49-70)	<0.01
Recipient primary disease			
COPD and emphysema	11 (37%)	11 (37%)	0.95
Cystic fibrosis and bronchiectasis	9 (30%)	9 (30%)	0.95
Fibrosing lung disease	6 (20%)	6 (20%)	0.95
Other	4 (13%)	4 (13%)	0.95
Ischemia time (hours) median (IQR)	5.6 (4.6-6.7)	6.3 (5.1-7.5)	0.055
Transport time (hours) median (IQR)	2.6 (2.0-3.2)	3.0 (2.3-3.7)	0.10
Total implant time (hours) median (IQR)	3.0 (2.2-3.8)	3.6 (2.6-4.3)	0.05

Abbreviations: COPD, chronic obstructive pulmonary disease; CMV, cytomegalovirus; CVA, cerebral vascular accident; ICH, intracerebral haemorrhage; IQR, interquartile range; RTA, road traffic accident; SS, senior surgeon; SSR, senior surgeon right lung.

Chi-square tests were used for categorical variables and *t-tests for continuous variables.*

In 30 cases, the operation was performed entirely by the SS. In another 30 operations, the senior surgeon implanted the right lung (SSR) and a trainee implanted the left lung (TL). Immediately after the surgery, the SS made a subjective assessment of their perceived workload by completing the NASA-TLX for the relevant phases of the procedure (SS or SSR and TL). The data were retrieved from the app, and frequency tables and graphs were generated. The Kolmogorov-Smirnov 2 sample test was used to evaluate the significance of results.

## Results

The mean scores for each of the subscales and the *p*-values from Kolmogorov-Smirnov 2 sample tests comparing SS with SSR and TL, and SSR with TL are shown in Table 2.

**Table 2:** Mean NASA-TLX Subcategory and Overall Weighted Scores with Corresponding *p*-Values.

Parameter	SS	SSR	TL	*p*-value SS vs SSR	*p*-value SS vs TL	*p*-value SSR vs TL
Mental demand	150.1	200.8	326.3	<0.001	<0.001	<0.001
Physical demand	146.2	136.8	264.7	0.799	<0.001	<0.001
Temporal demand	232.1	270.6	322.5	0.035	<0.001	0.001
Performance	253.1	268.5	263.8	0.586	0.799	0.953
Effort	282.5	285.3	311.2	0.953	0.035	0.134
Frustration	121.0	158.3	368.6	0.134	<0.001	<0.001
Overall weighted	32.3	95.0	68.8	<0.001	<0.001	0.007

Abbreviations: NASA-TLX, National Aeronautics and Space Administration Task Load Index; SS, senior surgeon; SSR, senior surgeon right lung, TL, trainee left lung.

**Figure 1:**
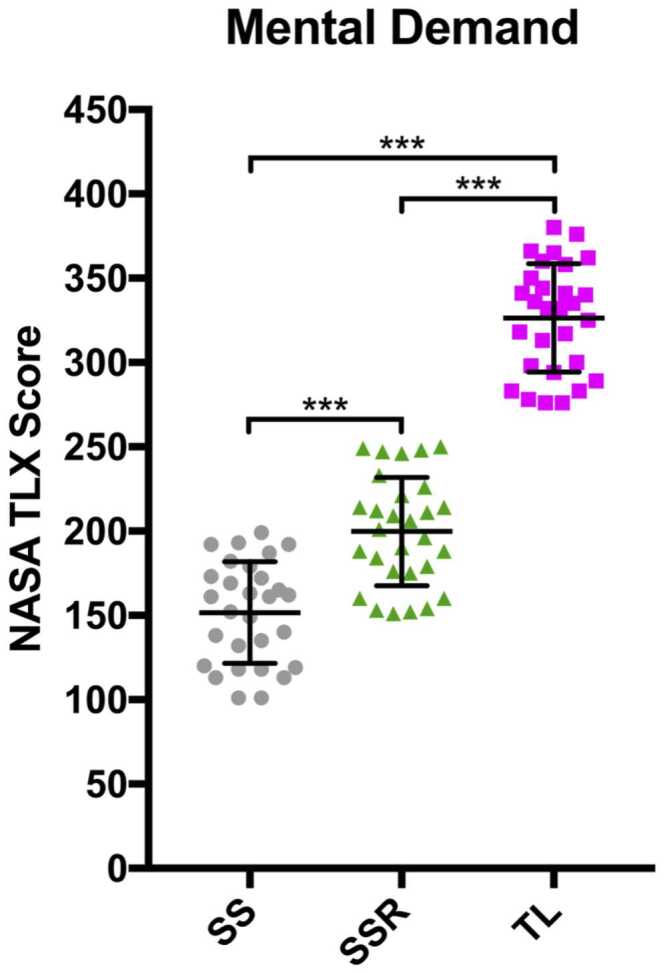
Graph comparing perceived mental demand NASA-TLX scores between SS, SSR, and TL (***p < 0.001). NASA-TLX, National Aeronautics and Space Administration Task Load Index; SS, senior surgeon; SSR, senior surgeon right lung; TL, trainee left lung.

The NASA-TLX scores for mental demand in each of the 3 surgeon configurations are presented in Figure 1.

Every data point for the trainee performing the left side was greater than those for SS and SSR. This was reflected in the Kolmogorov-Smirnov 2 sample test which showed mental demand was significantly greater (*p* < 0.001) when training, both while the trainee was operating and the SS performing the right side (SS mean 150.1; SSR mean 200.8; TL mean 326.3). Furthermore, mental demand for TL was also significantly greater than SSR (*p* < 0.001). Similar results were found for temporal demand (Table 2).

**Figure 2:**
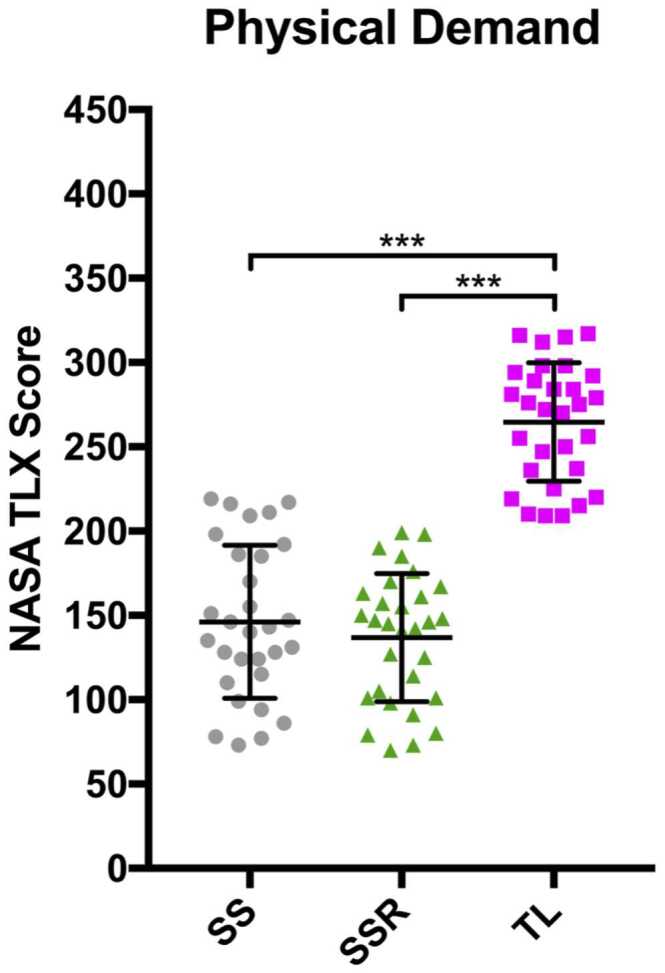
Graph comparing perceived physical demand NASA-TLX scores between SS, SSR, and TL (***p < 0.001). NASA-TLX, National Aeronautics and Space Administration Task Load Index; SS, senior surgeon; SSR, senior surgeon right lung; TL, trainee left lung.

The scores for physical demand are displayed in Figure 2. Scores while the trainee was operating were significantly greater (*p* < 0.001) than both SS and SSR (SS mean 146.2; SSR mean 136.8; TL mean 264.7). However, unlike mental and temporal demands, there was no significant difference between the surgeon performing the case themselves and the surgeon performing the right side during the teaching case. Similar results were found for frustration (Table 2). Perceived effort while the trainee implanted the left lung was only significantly greater than the SS cases (*p* = 0.035), with no significant difference to SSR (Table 2).

**Figure 3:**
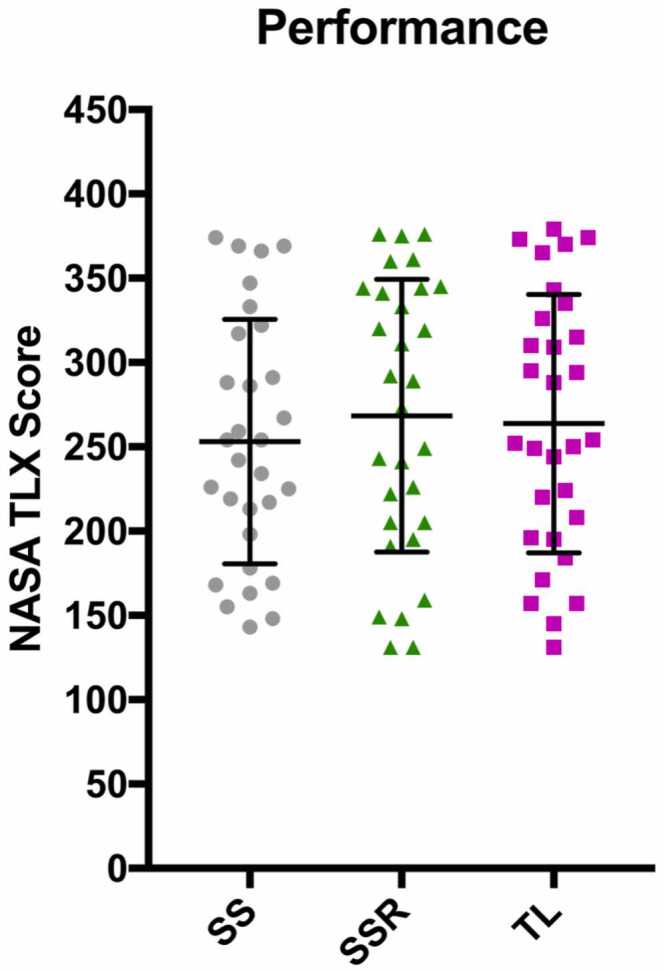
Graph comparing perceived performance NASA-TLX scores between SS, SSR, and TL. NASA-TLX, National Aeronautics and Space Administration Task Load Index; SS, senior surgeon; SSR, senior surgeon right lung; TL, trainee left lung.

The NASA-TLX scores for perceived performance showed considerable overlap between the 3 groups (Figure 3). Indeed, analysis confirmed no significant difference (*p* = 0.586/0.799/0.953) in these results (SS mean 253.1, SSR mean 268.5, TL mean 263.8).

**Figure 4:**
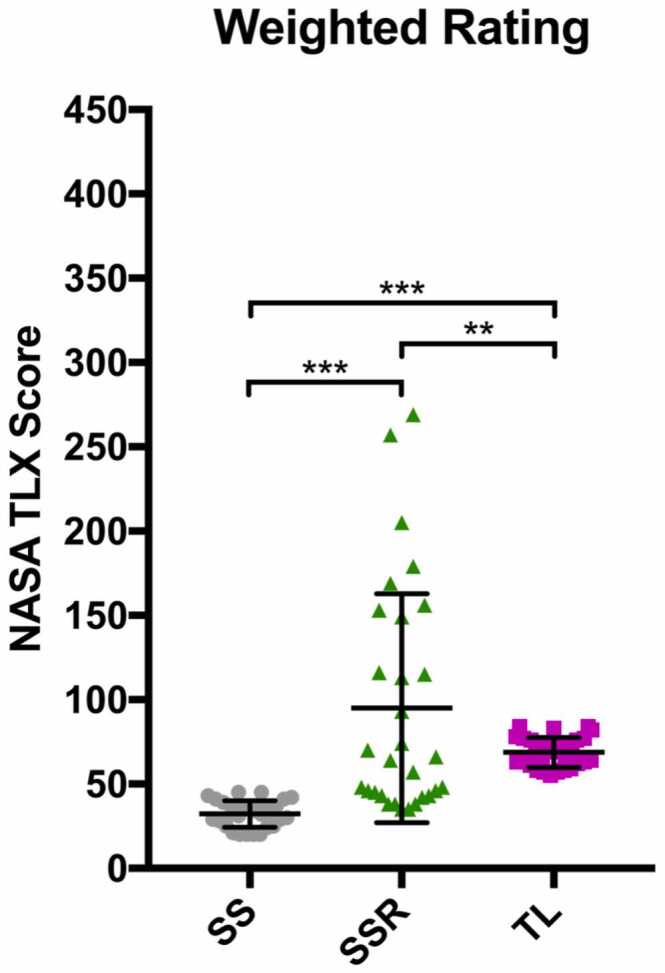
Graph comparing overall weighted NASA-TLX scores between SS, SSR, and TL (**p < 0.01, ***p < 0.001). NASA-TLX, National Aeronautics and Space Administration Task Load Index; SS, senior surgeon; SSR, senior surgeon right lung; TL, trainee left lung.

The weighted rating showed a clear difference in the distribution of scores, most noticeably between the SS alone and the trainee performing the left side (Figure 4, *p* < 0.001). The data for the trainer performing the right side were significantly higher than the other 2 scores; however, the data are more disparate due to a small number of more stressful cases.

## Discussion

There is little evidence for the effect that training in lung transplantation has on trainers. Often performed out of hours and demanding even when relatively straightforward,^2^ it is understandable that training is not always at the forefront of a transplant surgeon’s mind. Using the NASA-TLX,^3^ a subjective measure of perceived workload, we sought to quantify the impact of training trainees and draw a comparison with procedures where the SS performed the whole procedure.

The overall weighted score was significantly higher when training than when performing as the first operator and we suggest this will likely always be the case, even with the most proficient trainees. However, it is when we consider the individual subscales that we gain the most insight.

Although mental and temporal demands were significantly higher during all aspects of training, physical demand and frustration were only higher while the trainee was implanting the left, not while the SS did the right (Table 1). There was even less impact on effort. The operation was therefore not more demanding for the surgeon throughout the whole operation with regards to these latter aspects, just for the trainee’s portion. Crucially, there was no significant difference in perceived performance between the 3 sub groups. This is supported by data that our center has previously presented demonstrating no difference in complication rates between the side performed by the trainee and that by the SS.^30^ These conclusions are most pertinent and suggest that this should give transplant surgeons the confidence to train trainees.

Utilizing the NASA-TLX to monitor cognitive workload in lung transplantation has safety implications for patients. Evidence from studies in medicine has shown that there is a correlation between cognitive workload and poorer performance,^9^, ^31^, ^32^, ^33^ and also increasing likelihood of errors.^31^, ^32^ During their systematic review Dias et al found that several studies tried to quantify a cut-off, but concluded that further research is required to establish this “danger zone.”^9^ Even without an established limit, the NASA-TLX allows senior training surgeons to better understand their training style and the effect that training has upon them during long-duration complex operations with high-stakes outcomes. This enables surgeons to understand their physical and mental reactions in these situations and develop their skills as a trainer, mitigate against the effects on themselves, and increase the quality of training and therefore the safety of patients when teaching is taking place.

Building on this work, we feel that further analyses could include the effect of factors such as the time of the day, pathologies and health of recipients, and the learning stage of the trainee. This could provide more insight into the drivers that increase cognitive workload and influence training practice. Many studies in other professional practices have successfully used the NASA-TLX to monitor trainees during training and/or the learning curve.^11^, ^12^, ^13^, ^14^ In combination with trainers scores, this information could further influence decisions about readiness for training and allow tailored, trainee-specific development. Previous studies have suggested the NASA-TLX could be used to identify trainees likely to have a higher cognitive workload and hence more prone to error,^31^ allowing appropriate support to be provided. A final avenue for future exploration is incorporating real-time objective physiological methods, for example, monitoring heart rate, which would give greater weight than a subjective assessment alone. Correlation has previously been found between the NASA-TLX and blinking^34^ and also sympathetic tone.^13^ Furthermore, cardiac surgery training has been shown to significantly increase the heart rate of the supervising surgeon.^35^ Such methods could be integrated with the NASA-TLX to provide a more thorough assessment of workload, but must be balanced against any distraction the recording equipment may cause.

A final consideration is to place this study in the wider context of the evolving future of lung transplantation. Internationally the number of lung transplants performed annually continues to rise.^2^, ^36^ At the same time, the complexity of the procedures is increasing^2^ as we continue to make important advances,^37^ such as ex vivo perfusion^38^ and bridging to transplantation with extracorporeal membrane oxygenation.^39^ Recipients are therefore also potentially more sick, while at the same time there is a drive to use more marginal donor organs. Furthermore, due to an ongoing shortage of organs, measures to increase donation and the donor pool continue to develop, with the hope of seeing an increase in transplantation activity.^2^, ^37^ In the UK, a considerable proportion of the transplantation workforce is within 10 years of retirement, and from 2024, there is going to be a substantial need for recruitment of the transplant surgeons of the future.^2^ In the US, there are also concerns about meeting requirements, as 23% of surveyed lung transplant fellowship programs performed fewer transplants than the United Network for Organ Sharing minimum requirements and nationally the majority of fellows did not proceed to transplant appointments.^40^ It is therefore more important than ever that we are training the lung transplant surgeons of tomorrow with the highest quality of teaching and education in the operating room possible. By demonstrating in our center that training can be delivered without affecting perceived performance, we hope to give other surgeons the confidence to train trainees. Careful thought needs to be given to the design of training programs and we hope that tools such as the NASA-TLX could be informative and guide progress.

## Limitations

The subjective nature of the NASA-TLX combined with the length and complexity of lung transplant operations means that there is a risk of recall bias. Cases were evaluated immediately after the procedure with no delay, immediately after descrubbing. Potentially completing the NASA-TLX intraoperatively, for example, after the SS has completed the right side, may be one way to minimize this but could be impractical. Secondly, this was a single surgeon, single-center study, and therefore reflects on our specific unit training practice. Despite this, we hope to have demonstrated that this assessment tool can be applied to training in our surgical discipline. Given the subjective nature of the assessment, it is less likely that precise figures will be transferable across centers. Rather, the NASA-TLX can be employed as a method for individual trainers and trainees to monitor and review their own practice. Scores are likely to vary as trainees and SSs traverse the learning curve in their respective roles during the procedure.

In future iterations of our study, additional variables will need to be considered in an analysis framework encompassing larger numbers of patients, trainers, and trainees which were not formally considered for the purposes of this analysis. These would include the effect of day or nighttime operating, the better stratification of case complexities (degree of intrathoracic adhesions, patient anatomical factors, size mismatches), and how they affect the procedure's difficulty and the surgeon's mental strain. Decision making, perhaps at the time of weaning cardiopulmonary bypass or Extracorpeal Membrane Oxygenation support, is crucial in understanding the pressures and mental demands placed on surgeons during critical phases of the transplantation and needs to be better evaluated and understood. Incorporating these adjustments will significantly strengthen future studies using this evaluation tool in training in lung transplantation.

## Conclusion

The NASA-TLX can be used to inform the effects of training in lung transplantation on trainers. In particular, training leads to greater mental and temporal demands but has less effect on physical demand, frustration, and effort. Most importantly, we found no difference in perceived performance regardless of whether the trainer performed the entire procedure or implanted only the right lung and a trainee implanted the left.

These findings reflect favorably on the current model used for lung transplantation training. As the specialty continues to be confronted with diverse challenges, this study should give confidence to those training the transplant surgeons of the future, as well as provide them with a mechanism to reflect on their own training performance.

## Disclosure statement

None of the authors have any conflicts of interest, financial or otherwise. This research did not receive any specific grant from funding agencies in the public, commercial, or not-for-profit sectors.
